# Editorial: Bioactivity and health benefits of bee products: emerging technologies and consumer insights

**DOI:** 10.3389/fnut.2026.1786182

**Published:** 2026-02-03

**Authors:** Jailane de Souza Aquino, Silvia Valverde, Tatiana Colombo Pimentel

**Affiliations:** 1 Department of Nutrition, Health Sciences Centre, Federal University of Paraíba, João Pessoa, PB, Brazil; 2 Department of Agricultural Chemistry and Food Science, Universidad Autónoma de Madrid, 28049, Madrid, Spain; 3 Federal Institute of Paraná (IFPR), Paranavaí, Paraná, Brazil

**Keywords:** analytical strategies, bioaccessibility, biological assay, bioprocessing, healthy properties, sensory properties

The scientific and regulatory interest in bioactive compounds and nutraceuticals has expanded markedly in recent decades, driven by aligned governmental, industrial, and consumer-focused public policies aimed at promoting health, preventing chronic diseases, and supporting healthier and more sustainable food systems. Within this context, bee products, such as honey, propolis, royal jelly, bee pollen and related products are chemically complex matrices, rich in phenolic compounds, bioactive peptides, enzymes, and micronutrients and they have been used by humans for centuries with significant potential in modern nutrition, health and food sciences (1, 2).

Advances in analytical chemistry, food processing technologies, and experimental biology have substantially expanded knowledge on the chemical, nutritional, rheological, and sensory characteristics of bee products. In parallel, emerging evidence supports their antioxidant, anti-inflammatory, antimicrobial, and metabolic effects, reinforcing their potential role as dietary interventions or adjunctive strategies in disease prevention and treatment (3). However, despite these advances, important challenges remain, particularly regarding variability in composition, lack of universally accepted quality standards, and the need for validated methods that meet regulatory expectations for health claim substantiation and consumer protection, as well as for translating preclinical findings into clinically meaningful outcomes (4).

Beyond these scientific and regulatory considerations, the growing consumer interest in natural health-promoting ingredients and the increasing regulatory attention to nutraceuticals have reinforced the need to better characterize bee-derived products from a technological, safety, and efficacy standpoint. Bee products occupy a particularly dynamic interface between food and health, which amplifies both their commercial relevance and the need for standardized analytical, biological, and communication frameworks to ensure responsible innovation and consumer trust.

This Research Topic was conceived to integrate chemistry, technology, bioactivity, and consumer science within a translational framework aligned with European Food Safety Authority (EFSA)- and Food and Drug Administration (FDA)-oriented perspectives. The eight articles published in this Topic collectively address innovative strategies for the extraction, processing, characterization, and biological validation of bee products, while also considering sustainability and consumer response.

From a compositional and biodiversity perspective, *Unlocking the Bio-economic Potential of Native Non-Apis Bee Products in Pakistan* (Bodlah et al.) investigates the biochemical diversity of bee products derived from non-*Apis* species. This study highlights the influence of botanical origin and bee species on antioxidant capacity and phenolic profiles, emphasizing the economic and nutritional potential of underexplored bee products. The findings are particularly relevant for regions seeking to value local biodiversity while ensuring product quality and bioactivity.

At the molecular and cellular level, *Major Royal Jelly Proteins Promote C2C12 Myotubes Differentiation by Improving Mitochondrial Function* (Zhang et al.) provides mechanistic evidence supporting the biological activity of royal jelly proteins. Using an *in vitro* muscle cell model, the authors demonstrate improvements in mitochondrial function and myogenic differentiation, linking specific bioactive components to metabolic pathways. This study strengthens biological plausibility, a key criterion for translational nutrition and regulatory evaluation.

Technological innovation is a central theme in *Enhancing Antioxidant Activity and Quality of Triadica cochinchinensis Honey via an Automated Temperature-Humidity Controlled Cabinet* (Jiang et al.). This work demonstrates how controlled processing conditions can preserve or enhance the antioxidant properties of honey. By addressing processing-induced variability, the study contributes to standardization efforts essential for reproducibility, shelf stability, and regulatory compliance.

A broader technological and production-oriented perspective is provided in *Comprehensive Review on Improved Honey Production: Techniques, Challenges, Opportunities, and Future Prospects in Africa* (Tadele et al.). This review synthesizes current knowledge on honey production systems, highlighting technological gaps, environmental challenges, and opportunities for quality improvement. The article underscores the importance of harmonizing production practices with quality standards to ensure safety, consistency, and market competitiveness.

The translational relevance of bee products for metabolic health is addressed in *The Protective Effect of Saudi Arabian Bee Honey Against Excessive Weight Gain and Obesity-Related Parameters in Rats Fed a High-Fat Diet* (Tamim et al.). Using an *in vivo* model, this study demonstrates that honey supplementation mitigates weight gain and metabolic alterations induced by a high-fat diet. These findings support the potential role of honey as a dietary component in metabolic regulation, while also highlighting the need for dose–response and human studies.

Similarly, *Malícia Honey (Mimosa quadrivalvis L.) Produced by the Jandaíra Bee (Melipona subnitida D.) Shows Antioxidant Activity via Phenolic Compound Action in Obese Rats* (Bezerra et al.) focuses on stingless bee honey and its phenolic-driven antioxidant effects in an obesity model. The study reinforces the importance of botanical and entomological origin in determining biological activity and supports the inclusion of non-*Apis* honeys in functional food research.

Finally, *In Vitro Testing of Honey Quality and Biological Functionality: Underestimated Elements in the Clinical Testing of Honey* (Majtan) critically discusses analytical and biological testing approaches, underscoring the importance of validated *in vitro* models for supporting clinical relevance and regulatory acceptance of honey-based products.

Taken together, the articles in this Research Topic illustrate how chemistry, emerging processing technologies, and biological assays are converging to support the scientific and market maturation of bee products. Nevertheless, the field still faces important challenges, including the need for cross-laboratory standardization, improved dose—response characterization, deeper mechanistic understanding, and human studies capable of informing regulatory and clinical decision-making. Sustainability and biodiversity also emerge as strategic dimensions, especially for non-*Apis* species and nest-derived biomaterials, which require coordinated frameworks for valorization and conservation.

In summary, the eight articles published in this Research Topic collectively demonstrate how advances in chemistry, technology, bioactivity assessment, and consumer-oriented research can enhance the scientific and regulatory understanding of bee products. By integrating compositional characterization, biological validation, sustainability, and quality parameters, these contributions strengthen the translational relevance of bee-derived foods and nutraceuticals. Future research should continue to prioritize standardization, bioavailability, and clinical validation to support responsible innovation and regulatory decision-making.
